# Genome-wide analysis of the GRAS gene family in *Liriodendron chinense* reveals the putative function in abiotic stress and plant development

**DOI:** 10.3389/fpls.2023.1211853

**Published:** 2023-09-21

**Authors:** Yuhao Weng, Xinying Chen, Zhaodong Hao, Lu Lu, Xinru Wu, Jiaji Zhang, Jingxiang Wu, Jisen Shi, Jinhui Chen

**Affiliations:** ^1^ State Key Laboratory of Tree Genetics and Breeding, Co-Innovation Center for Sustainable Forestry in Southern China, Nanjing Forestry University, Nanjing, China; ^2^ Key Laboratory of Forest Genetics and Biotechnology of Ministry of Education, Nanjing Forestry University, Nanjing, China

**Keywords:** *Liriodendron*, GRAS gene family, phylogenetic analysis, abiotic stress, phytochrome A signal transduction, transiently overexpressed

## Abstract

**Introduction:**

*GRAS* genes encode plant-specific transcription factors that play essential roles in plant growth and development. However, the members and the function of the *GRAS* gene family have not been reported in *Liriodendron chinense*. *L. chinense*, a tree species in the *Magnolia* family that produces excellent timber for daily life and industry. In addition, it is a good relict species for plant evolution research.

**Methods:**

Therefore, we conducted a genome-wide study of the *LcGRAS* gene family and identified 49 *LcGRAS* genes in *L. chinense*.

**Results:**

We found that *LcGRAS* could be divided into 13 sub-groups, among which there is a unique branch named HAM-t. We carried out RNA sequencing analysis of the somatic embryos from *L. chinense* and found that *LcGRAS* genes are mainly expressed after heart-stage embryo development, suggesting that LcGRAS may have a function during somatic embryogenesis. We also investigated whether GRAS genes are responsive to stress by carrying out RNA sequencing (RNA-seq) analysis, and we found that the genes in the PAT subfamily were activated upon stress treatment, suggesting that these genes may help plants survive stressful environments. We found that PIF was downregulated and COR was upregulated after the transient overexpression of PATs, suggesting that PAT may be upstream regulators of cold stress.

**Discussion:**

Collectively, *LcGRAS* genes are conserved and play essential roles in plant development and adaptation to abiotic stress.

## 1 Introduction

The GRAS gene family is a large plant-specific transcription factor family that is indispensable in many biological processes. It responds to hormonal signals, such as Gibberellins (GA) and (Jasmonic acid) JA. Some GRAS genes have functions in meristem maintenance and root development in plants (Bolle, 2004). It has been more than 20 years since the first three *GRAS* genes, *GIBBERELLIC ACID INSENSITIVE (GAI)* (Peng et al., 1997), *REPRESSOR OF GA1 (RGA)* (Silverstone et al., 1998), and *SCARECROW (SCR)* (Di Laurenzio et al., 1996), were discovered. The name of this transcription factor family is derived from these three members. In *Arabidopsis thaliana*, the GRAS gene family is divided into eight subfamilies (Lee et al., 2008), named DELLA, LAS (lateral suppressor), SCR (scarecrow), SHR (short-root), PAT1 (phytochrome A signal transduction 1), HAM (hairy meristem), LISCL (*Lilium longiflorum* scarecrow-like), and SCL (scarecrow-like), which is the same as in rice (*Oryza sativa* L. ssp. japonica) (Tian et al., 2004). The number of subfamilies is different across species: eight subfamilies in *Brassica rapa* ssp. pekinensis (Song et al., 2014); 10 subfamilies in *Isatis indigotica* (Zhang et al., 2016); 11 subfamilies in *Prunus mume* (Lu et al., 2015); 13 subfamilies in *Brassica napus* (Guo et al., 2019) and *Solanum lycopersicum* (Huang et al., 2015); 16 subfamilies in *Medicago truncatula* (Song et al., 2017); and 17 subfamilies in *Amborella trichopoda* (Cenci and Rouard, 2017). There are still no standard criteria to classify the subfamilies of GRAS so far. The minimum commonality of the GRAS members is that all genes should have a highly conserved C-terminus with two leucine heptad repeats (LHRI and LHRII) (Pysh et al., 1999) and a highly divergent N-terminus that may contain activation domains. A reasonable delineation may help the study of gene functions.

The GRAS gene family is involved in GA and auxin signaling pathways, phytochrome signaling pathways, and stress-induced signaling pathways (Khan et al., 2022). DELLA proteins, the most intensively studied subfamily, are well known as a key factor in the GA signaling pathway. GA-GID1-DELLA is the core regulatory module for sensing and responding to GA content under multiple endogenous signals and environmental cues (Sun, 2011). In brief, with an increasing GA level, GID1 changes the conformation to form the GA-GID1-DELLA complex. The DELLA proteins are proteolyzed via the 26S proteasome after being polyubiquitinated. It releases the downstream genes and prevents them from being repressed by the DELLA protein (Wang and Deng, 2014). In addition, they not only negatively regulate GA signaling but also physically interact with other GRAS members. In *M. truncatula*, DELLA forms a protein complex with NSP2 (Jin et al., 2016) and NSP1, which belong to HAM and SHR subfamilies, respectively (Zhang et al., 2017).

The GRAS family plays important roles in the development and maintenance of shoot apical meristem (SAM), root apical meristem, and axillary meristem (Sun et al., 2012). For example, in *Petunia hybrida*, HAM acts non-cell-autonomously to maintain the shoot apical meristem (Stuurman et al., 2002). In *Arabidopsis*, SHR is required for the asymmetric cell division of endodermis in roots and regulates root and shoot radial patterning upstream of SCR (Helariutta et al., 2000). SCR is expressed in the quiescent center to retain the stem cell’s identity (Sabatini et al., 2003). LAS is indispensable for the initiation of axillary meristems at a distance to SAM (Greb et al., 2003), particularly during the vegetative phase. RAM is one of the GRAS subfamilies, and it is critical for the induction of hyphopodia formation in AM (arbuscular mycorrhizal) fungi (Gobbato et al., 2013). SCL plays a role in the very early stages of adventitious root formation in auxin- and stress-induced signaling in *Pinus radiata* and *Castanea sativa* (Sanchez et al., 2007). For example, SCL14 could interact with the Class II TGA transcription factor TGA/SCL14 complex. It activates the transcription of the corresponding genes that are involved in a detoxification network (Fode et al., 2008). In rice, *OsSCL7*, a member of the SCL4/7 subfamily, has a function in disease resistance and plant immunity (Lu et al., 2022). LISCL is another subfamily that differs from SCL. It was first reported from lily (*Lilium longiflorum*) (Morohashi et al., 2003) that *LISCL* is involved in the transcriptional regulation of microsporogenesis (Morohashi et al., 2003). More importantly, *AtSCL13* and *PAT1*, as members of the PAT subbranch, are involved in phytochrome signaling (Torres-Galea et al., 2006) and negatively regulate shoot branching (Muntha et al., 2019). Most PATs are related to phytochrome A signaling, but *SCL13* is also involved in phytochrome B signaling (Torres-Galea et al., 2006). In *Arabidopsis*, the mutant of *pat* shows strongly reduced responses in continuous far-red light (Bolle et al., 2000). In *Brassica juncea*, *PAT1PAT1* interacts with *CONSTANS-LIKE 13* and *BRC1* to mediate bud outgrowth, shoot branching, and flowering (Muntha et al., 2019).

The identification of the GRAS gene family is increasingly focused on cash crops, including rice, tomato, pepper (*Capsicum annuum* L.) (Liu et al., 2018), pecan (*Juglans regia*) (Quan et al., 2019), apple (*Malus domestica*) (Fan et al., 2017), maize (*Zea mays* L.) (Guo et al., 2017), tea plant (*Camellia sinensis*) (Wang et al., 2018), and cotton (*Gossypium hirsutum*) (Zhang et al., 2018). Research on this family in woody plants is still very limited. *Liriodendron* is an important woody and landscape species with ornamental, economic, and ecological value (Guan et al., 2021). In addition, it is a great species for studying plant evolution because of its unique evolutionary status, which is situated between the basal angiosperm and monocots or eudicots (Chen et al., 2019). In different hybrid *Liriodendron* genotypes, seedlings derived from somatic embryos vary substantially in root development, which determines the survival rate of seedling transplantation and the efficiency of tree breeding. Since GRAS genes regulate root radial patterning, the investigation of the GRAS gene family in *L. chinense* is the first step for us to understand root formation during somatic embryogenesis in *Liriodendron*. Therefore, we carried out a genome-wide analysis of the GRAS gene family in *L. chinense*. Our results provide insights into the function of the GRAS gene family, which will help prove the efficiency of *Liriodendron* breeding.

## 2 Materials and methods

### 2.1 Genome-wide identification of LcGRAS genes

The latest version of the sequence of genome DNA, CDS (coding sequence), and the protein of *L. chinense* was downloaded from CNGBdb (CNA0002404, China National GeneBank DataBase). We downloaded GRAS family HMM (hidden Markov model) profile PF03514 from the Pfam database (https://pfam.xfam.org) to search candidate GRAS genes by using HMMER 3.3. Furthermore, we used the *de novo* transcriptome data of somatic embryos in hybrid *Liriodendron* (data not published) to screen candidate GRAS genes and map them with the results. The protein sequences of candidate GRAS genes were uploaded to the NCBI protein database, which was used as initial queries by BLASTp for further verification. Finally, we checked the sequence and annotation by using the online Softberry software (https://www.softberry.com/) and CD-search (https://www.ncbi.nlm.nih.govStructure/cdd/cdd.shtml). In addition, we used the online EXPASY software website (https://www.expasy.org/) to analyze the theoretical pI (isoelectric point of protein), molecular weight, and other basic analyses of the GRAS genes’ information.

### 2.2 Analysis of the protein conserved motif and the cis element in the promoter

We used the MEME (https://meme-suite.org/meme/tools/meme) (version 5.3.3) program to identify the conserved motifs of *LcGRAS* using the classic mode. We increased the minimum width to 10 and increased the number of motifs to 10. We extracted the 2,000-bp length of the DNA sequence, which is upstream from every *LcGRAS* gene, as the promoter sequence to analyze the cis element by using PlantCARE (http://bioinformatics.psb.ugent.be/webtools/plantcare/html/). Then, repeated motifs or motifs without explicit functional annotations in the results were manually deleted and sorted by function.

### 2.3 Phylogenetic analysis of the GRAS gene family in different species

We aligned the protein sequences of LcGRAS using Clustal Omega. A phylogenetic tree was constructed via the NJ (neighbor-joining) method with 1,000 bootstrap replicates using MEGA7. To further understand the classification of the GRAS gene family, we downloaded the GRAS protein sequences of other several species from PlantTFDB (http://planttfdb.gao-lab.org/), including *A. thaliana*, *A. trichopoda*, *O. sativa*, *Picea abies*, and *Populus trichocarpa*. The genomic data of *Euryale ferox* were downloaded from CoGe (https://genomevolution.org/coge/). *EfGRAS* was identified by HMM, and the blast was the same as *LcGRAS*. After alignment, the GRAS data of *Magnolia biondii* were available in the Dryad database: https://doi.org/10.5061/dryad.s4mw6m947. RaXml (Stamatakis, 2014) was used to construct the ML (maximum likelihood) tree with 1,000 bootstraps, and it was drawn using the online iTOL tool (https://itol.embl.de/). The Ka/Ks of the GRAS gene was calculated using KaKs_calculator 2.0.

### 2.4 Chromosomal distribution and gene duplication analysis

We obtained genomic data at the chromosome level in *L. chinense* internally. Because some contigs are still not assembled into chromosomes, we merged all remaining contigs that were not assembled according to the serial number. Each contig was segmented into a 50-bp N base. The final sequence was named chrF (chr-Fake). MCScanX was used to find out the collinearity and duplication relationship among genes in *L. chinense*, and the results are shown using Circos (Krzywinski et al., 2009). RIdeogram (Hao et al., 2020) was used to create a figure for the collinearity analysis carried out on four species.

### 2.5 Plant preparation, transformation, and stress treatments

The materials of somatic embryogenesis were collected sequentially, starting with collecting pre-embryonic mass (PEM) and then collecting materials from the small plant stage (PL); the materials were collected from a somatic embryogenesis system (Li et al., 2012). In detail, the embryogenic callus with pro-embryogenic cell mass (PEM) was cultured in the induced media after the preculture for 10 days (ES1). The cells after sieving were named ES2. We collected the samples that were cultured in the induced media at the first and third day named ES3 and ES4, respectively. Globular embryos could be observed approximately 7 days that we collected the globular embryos as the third sample named ES5. And then, we collected heart-shaped embryos (ES6), torpedo-shaped embryos (ES7), immature cotyledonary embryos (ES8), mature cotyledonary embryos (ES9), and little plant (PL) in proper order. Each sample has three independent biological replications. The plants of hybrid *Liriodendron*, which regenerate from somatic embryos, were cultivated for 2 months in 3/4 MS medium. All plants were cultivated in a room containing tissue culture under white light (light for 16 h; darkness for 8 h). The transient genetic transformation method used is the same as the method used in *Tamarix hispida* (Zang et al., 2017). We collected whole plants 48 h after transformation. Based on the limitation of the genetic transformation system, only the first mature leaves have high transgenic efficiency. Therefore, only apical leaves were collected after the transformation. Additionally, three independent biological replicates were prepared for later expression validation. To simulate cold stress, plants were held at 4°C for 12 h, 24 h, and 48 h. To simulate heat and drought stress, plantlets were held at 40°C or subjected to a 15% PEG 6000 treatment for 1 h, 3 h, 6 h, 12 h, 1 day, and 3 days. We collected leaves as samples with three independent biological replicates. All samples were frozen in liquid nitrogen immediately and then stored at −80°C for RNA extraction by RNA-seq analysis and quantitative real-time PCR (qRT-PCR) experiments.

### 2.6 The LcGRAS expression analysis under stress treatments and in different tissues

We downloaded transcriptome data from NCBI (PRJNA679101 and PRJNA679089). We obtained the expression data of *LcGRAS* in treatments with cold (raw data unpublished), hot, and drought (**Supplementary Table S3**) conditions and during somatic embryogenesis (raw data unpublished) (**Supplementary Table S4**). The transcriptome data of different tissues are shown in **Supplementary Table S5**. We used R package (pheatmap) to make the heatmap. Total RNA was extracted from each sample by using a FastPure Plant Total RNA Isolation Kit (Vazyme). HiScript 1st Strand cDNA Synthesis Kit (Vazyme) was used for first-strand cDNA synthesis. We used the AceQ qPCR SYBR Green Master Mix (Vazyme) to carry out qRT-PCR analysis in the LC480 platform. The primers used in this study are listed in **Supplementary Table S4**. We performed three biological replicates for each gene. The gene named *Tubulin* was used as the reference gene. The relative expression levels were calculated using 2-ct.

## 3 Results

### 3.1 Genome-wide identification of the GRAS gene family in *L. chinense*

We performed a genome-wide HMM search and homology analysis to obtain putative GRAS genes by using all protein sequences from the reference genome of *Liriodendron*. We kept the putative targets when the E-value was less than 1e-5. The *de novo* transcriptome of *Liriodendron* was used to confirm that no possible genes have been missed. We further confirmed that all candidates have the right domains or motifs and reannotated the genes by using the online FGENESH software and Conserved Domain Search Service (CD Search) in Softberry and NCBI, respectively. In total, 49 genes were considered as GRAS genes in the *L. chinense* with a typical GRAS domain and complete gene structure (**Supplementary Table S1**). The length of the majority of proteins was 400–700 amino acids, but a few of them were longer than 800 amino acids. Protein Lc34435, the longest gene in all GRAS genes in *L. chinense*, is 995 amino acid-long.

**Figure 1:**
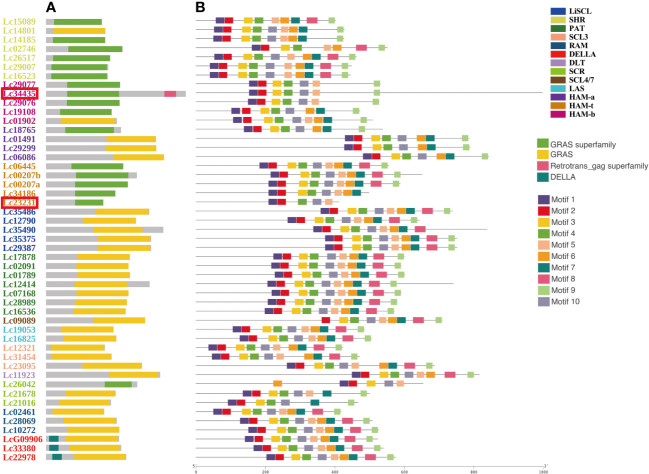
The conserve domains and conserved protein motifs of 49 *LCGRAS*. The genes were ordered by the subfamilies and distinguished by color. The subfamily name was annotated in the upper left corner. The two special genes, *Lc34435* and *Lc23231*, were marked in red box. **(A, B)** The distribution of domains and motifs on the lines in the graph represents their relative positions and lengths on the complete LCGRAS protein sequences. The different domains and motif are distinguished by color and annotated in the left of figure. The specific sequences of motifs are shown below. (Motif 1: RVHIIDFDIMQGLQWPTLMQALAARP; Motif 2: AYHAFYEICPYLKFGHFTANQAILEA; Motif 3: EIVNIIACEGAERVERHERLGKWRARMGRAGF; Motif 4: BHNGPSFLTRFLEALHYYSALFDSLE; Motif 5: KQJRQLSSPFGDPMQRLAAYFAEAL; Motif 6: ERLZETGRRLADFARSL GIPFEFHPV; Motif 7: GCLLLGWKGRPLVSASAWK; Motif 8: PNPRDSLLRMIRSLNPKVVTVVEQEA; Motif 9: VDLVQLLJACAEAVSAGBLELARALL; Motif 10: EMLKVRDGEALAVNCVLQLHHM).

**Figure 2:**
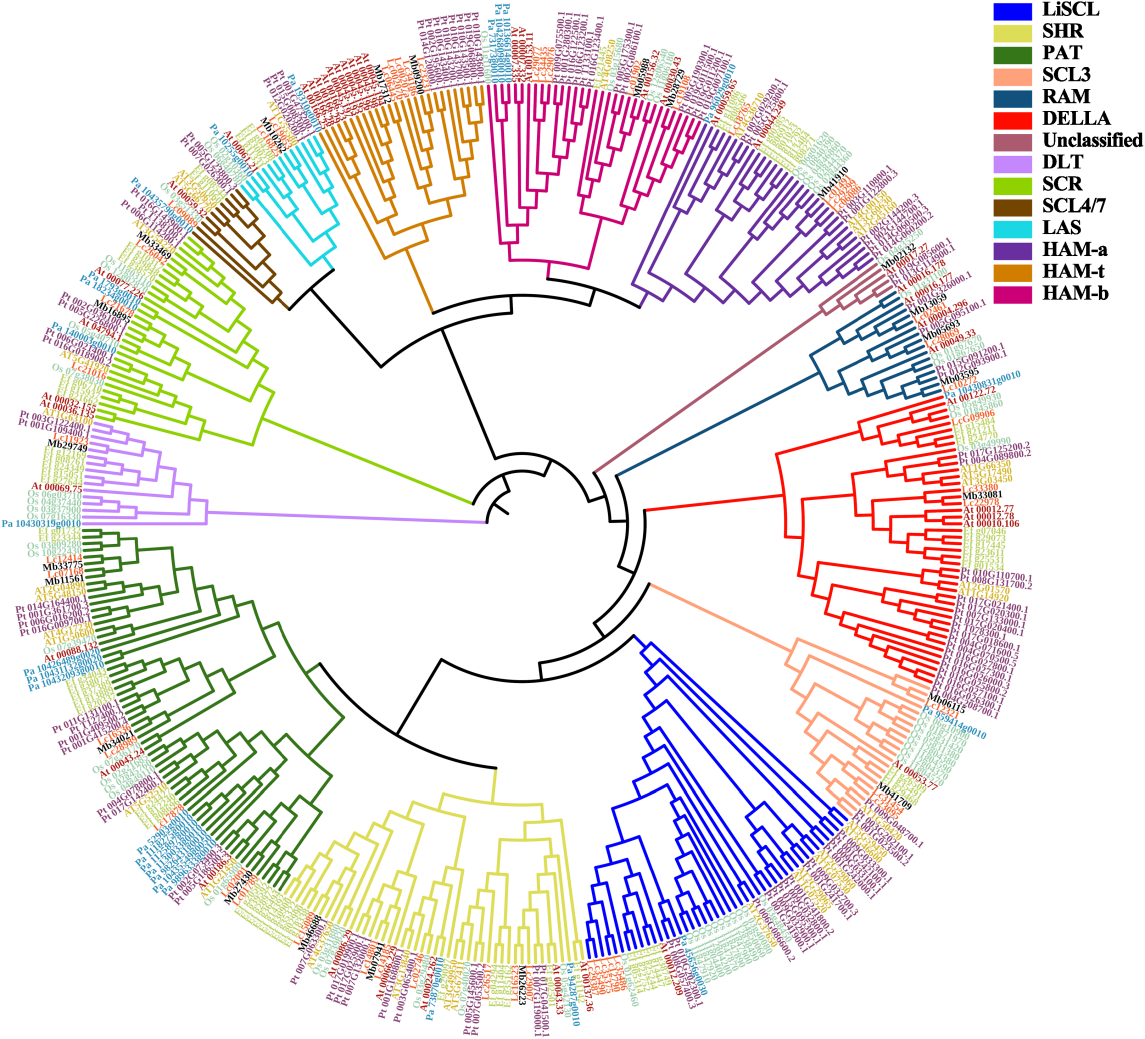
Phylogenetic relationships of GRAS proteins from 7 different plant species. The gene ID start with ‘Lc’ represents *L. chinense*; ‘AT’ represents *A. thaliana*; ‘At’ represents *A. trichopoda*; ‘Ef’ represents *E. ferox*; ‘Mb’ represents *P. trichocarpa*, ‘Pt’ represents *P. trichocarpa*; ‘Os’ represent *O. sativa*; ‘Pa’ represent *P. abies*, respectively. And the genes’ names were marked as different color to help distinguish the species. LCGRAS were marked as orange.

The GRAS genes do not have a complex domain structure. All have a representative domain named GRAS (pfam03514) or GRAS superfamily (cl15987). Notably, the DELLA subfamily is named based on its extra DELLA (pfam12041) domain in the N-terminal (**Figure 1A**). We searched the conserved motifs of all GRAS proteins by using MEME via the classic mode. Almost all motifs were located on the GRAS domain or GRAS superfamily (**Figures 1A, B**). Overall, 10 motifs were present for most genes. However, based on the results of subfamily classification (**Figure 2**), we found that some motifs help in the identification and functional characterization of GRAS subfamilies. For example, only *Lc09089* missed Motif 1. Motif 1 may be an element for identifying SCL4/7. Motif 8 was missing in SCR, HAM-a, and HAM-b. HAM-a also missed Motif 5, and SCR also missed Motif 6 (**Figure 1B**).

It is worth noticing that only *Lc34435* has a domain named the Retrotrans_gag superfamily (cl29674) (**Figure 1A**), which does not exist in any GRAS gene. However, *Lc34435* has a standard and representative domain named the GRAS superfamily, which is unique to the GRAS genes. Moreover, *Lc34435* is a highly homologous relative to other LcGRAS proteins. For these reasons, we kept this gene as a member of the LcGRAS family. *Lc23231* is a structurally incomplete GRAS protein with a shorter gene length and an incomplete domain in the C terminus (**Figure 1**). We found that there are similar structurally incomplete genes in the GRAS family in *A. thaliana* and other species as well. However, when aligning the protein sequences from these several species, we found that they do not share any motifs (**Supplementary Figure S1**). Since this is a common phenomenon for the GRAS family in different species, we kept this gene for the following analysis.

### 3.2 Duplication and cis-regulatory element analysis

**Figure 3:**
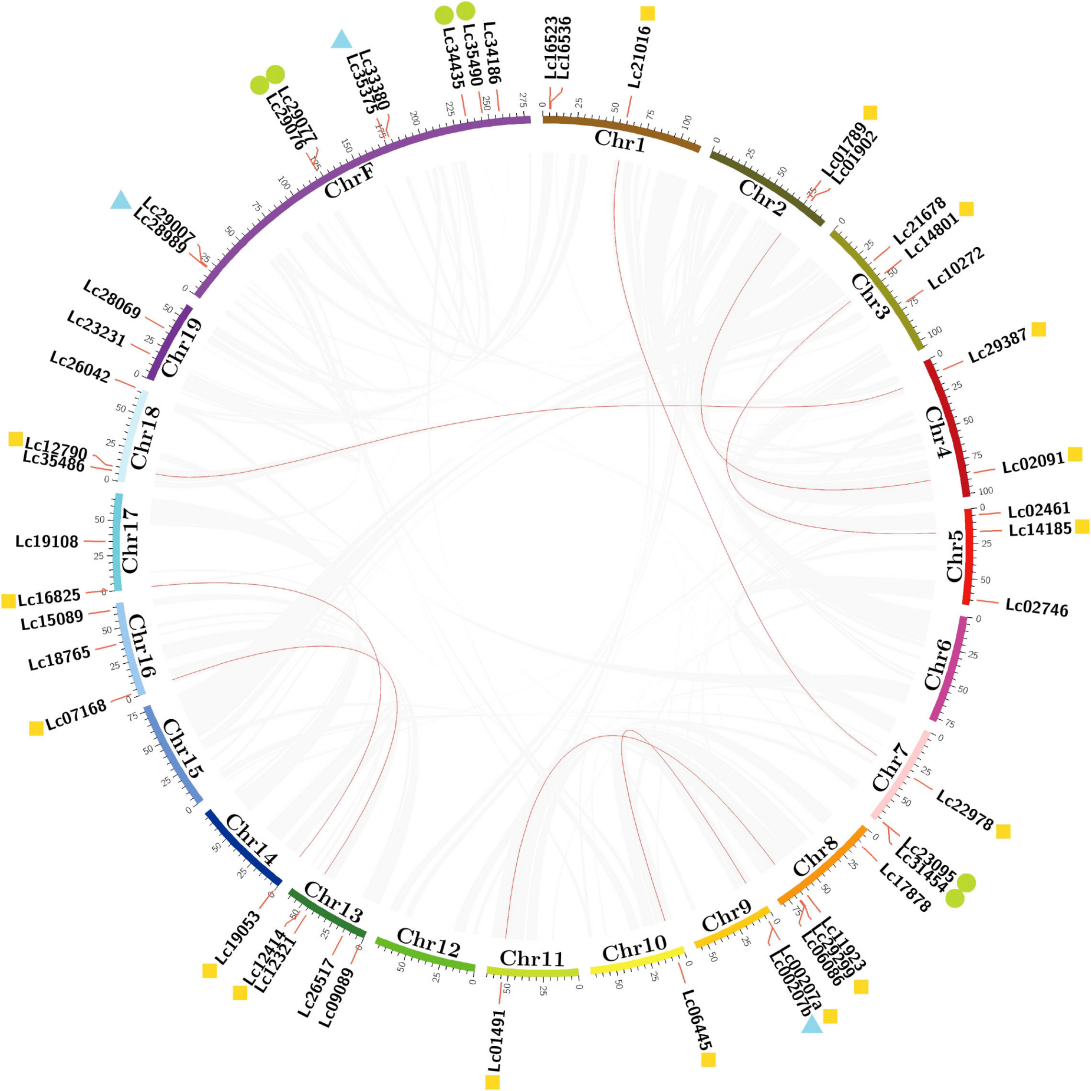
Schematic representations of the inter-chromosomal relationships of LCGRAS genes. Gray lines indicate all syntenic blocks in the *L. chinense* genome, and red lines indicate systematic gene pairs of LCGRAS. The yellow marker means WGD; green markers mean proximal duplications; blue markers mean tandem duplications.

*LcGRAS* was distributed on the chromosomes except for Chr6, Chr12, and Chr15 (**Figure 3**). We found that some genes are highly identical in terms of motif and domain distribution, such as *Lc29387* and *Lc35375*. They have the same length with respect to coding sequences and only a two-nucleotide difference. It might be derived from a duplication event. To find out all possible duplication genes, we used MCScanX to calculate the collinearity relationship in *LcGRAS* (**Figure 3**). There are a total of 16 genes belonging to WGD (whole-genome duplications), which form eight pairs. In addition, three couples of tandem repeats and six genes of proximal repeats were found in Chr7, Chr9, and ChrF. Duplication may be a cause for the increased number of *LcGRAS* family members.

Cis-acting regulatory elements (CREs) are closely related to gene function regulation, and their divergence is a common cause of evolutionary change (Wittkopp and Kalay, 2012). To address what could be the upstream regulators, we used a 2,000-bp-length sequence upstream of each gene to search for CREs by using PlantCARE software. Except for common CREs such as A-box and TATA-box, CREs are related to regulated hormones, stress responsiveness, light responsiveness, development, and MYB (**Supplementary Figure S2**). Light responsiveness and MYB-binding sites have the greatest number of all CREs. Except for the GT1 motif and G-box, there were 24 different CREs related to light responsiveness. This suggests that *LcGRAS* may be intensively regulated by the light signal. Furthermore, *LcGRAS* may be under hormone regulation. The CREs, such as AuxRR-core, ABRE, P-box, O2-site, TCA-element, as-1, and ERE, were related to auxin, ABA, GA, zein, salicylic acid, MeJA, and ethylene, respectively. W-box, WRE3, WUN motif, and STRE are CREs that are involved in wound or stress responsiveness. MBS is an MYB-binding site involved in drought inducibility, and LTR is involved in low-temperature responsiveness. This suggests that *LcGRAS* may have functions when plants are under stress, such as cold or drought conditions, and MYB may potentially be a regulator of *LcGRAS*.

### 3.3 Phylogenetic analysis of the GRAS gene family

To explore the phylogenetic relationship of *GRAS* and classify the subfamily of *LcGRAS*, we include the GRAS gene family from *A. thaliana*, *A. trichopoda*, *O. sativa*, *P. abies*, *P. trichocarpa*, and *E. ferox* to construct the phylogenetic tree. Firstly, we identified 67 *GRAS* genes in *E. ferox* by using the blastp of NCBI and HMM, which is the same as the work carried out on *L. chinense* (**Supplementary Figure S3**). *E. ferox*, which belongs to magnoliids, is used to support the phylogenetic status of *L. chinense*.

In total, 385 genes were divided into 14 clusters (**Figure 2**). One of 14 clusters, which contain *Os_04g35250*, *At_00017.27*, *Pt_019G085600.1*, and *Pt_013G114900.1*, was named as unclassified. This is because these four genes independently form a branch and cannot be properly clustered into any known clade. HAM genes were separated into three subclades, and this separation was based on whether they have Motif 5 and Motif 8 or not (**Figure 1B**). Notably, it is interesting that the members of cluster HAM-t all came from woody plants.

Next, based on subfamilies, we calculated Ka and Ks for each LcGRAS gene with genes in the same subfamily. The Ka/Ks value of gene pair *Lc29387* and *Lc35375* is expected to be 49.4, and the other gene pairs belong to neutral evolution or purifying selection (**Supplementary Figure S4**). The resulting p-value of *Lc29387* and *Lc35375* is approximately 0.375. Since these two genes are extremely similar to each other and only have two SNPs (single nucleotide polymorphism), evidence that these two genes belong to positive selection is insufficient. In general, the gene pairs in *Liriodendron* have a lower non-synonymous substitution rate, suggesting that the genes in the same subfamily may not have strong functional divisions.

**Figure 4:**
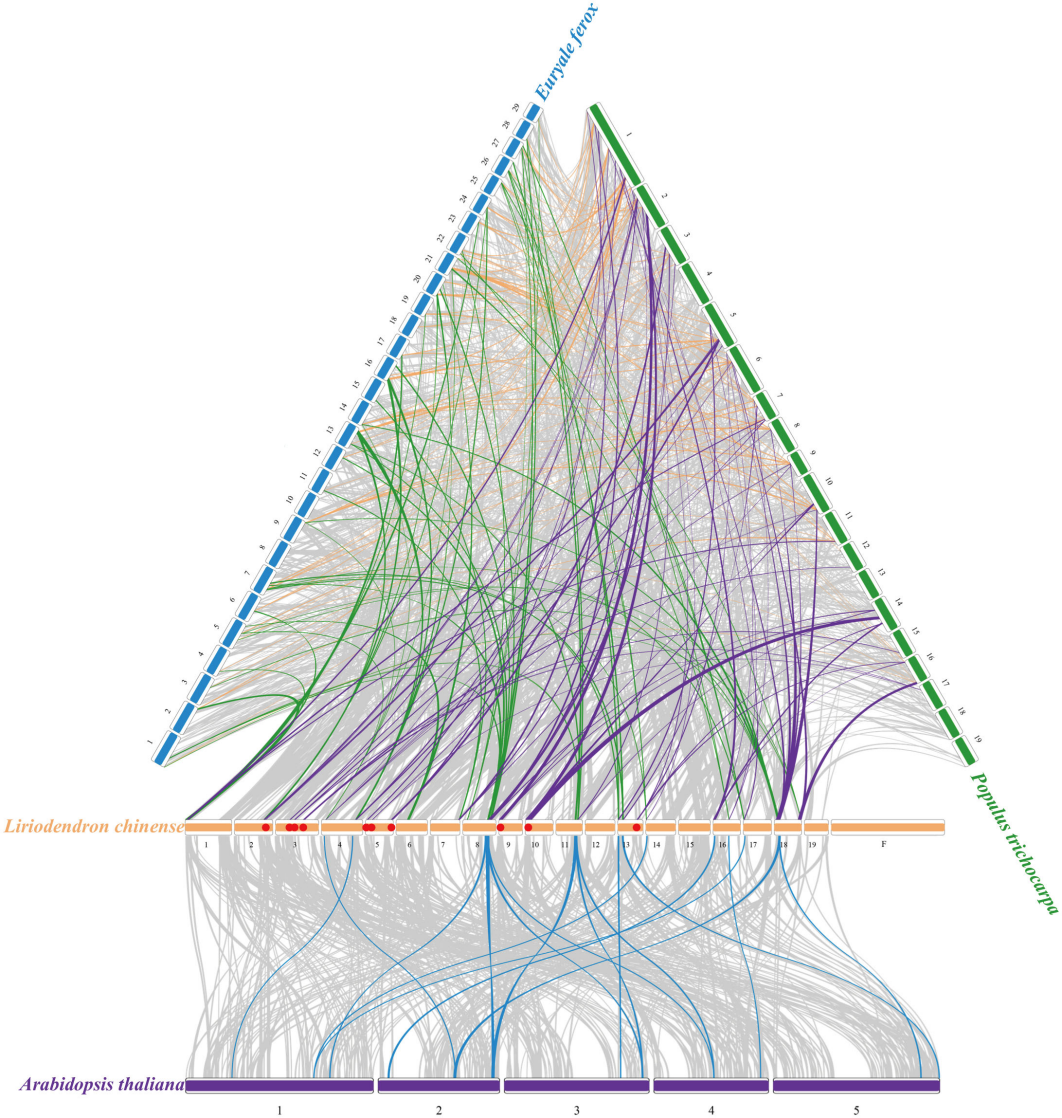
Syntenic analysis of *L. chinense* genes in comparison with those in 3 plant species (*A. thaliana, E. ferox, P. trichocarpa*). Grey lines mean the collinearity region between species, and the colorful line mean the collinearity region contained the LCGRAS. The red dots represent the unique syntenic gene pairs of *L. chinenes* and *P. trichocarpa*.

To further address the phylogenetic relationship with other species, we constructed four synteny maps containing *L. chinense*, *A. thaliana*, *E. ferox*, and *P. trichocarpa*. A total of 34 *LcGRAS* genes showed a syntenic relationship with these species (**Figure 4**). It is obvious that *LcGRAS* had the most syntenic gene pair with respect to *P. trichocarpa* (63 pairs) and the least syntenic gene pair with respect to *A. thaliana* (17 pairs). We found that the unique syntenic gene pairs of *L. chinense* and *P. trichocarpa* have an independent clade of the phylogenetic tree, which only consisted of genes from woody plants, such as *Lc00207a* (*HAM-t*), *Lc06445* (*HAM-t*), *Lc14185* (*SHR*), and *Lc14801* (*SHR*). The function of these genes is closely related to the growth, development, and morphogenesis of trees. Moreover, the genes, such as *Lc01491* (*HAM-a*), *Lc29299* (*HAM-a*), *Lc18765* (*HAM-a*), *Lc11923* (*DLT*), *Lc02091* (*PAT*), *Lc09089* (*SCL4/7*), *Lc16825* (*LAS*), *Lc19053* (*LAS*), *Lc26517* (*SHR*), *Lc12790* (*LiSCL*), and *Lc29387* (*LiSCL*), had syntenic genes with *GRAS* genes in all three species, suggesting an important role of these genes in evolution, and they exhibit more basic functions in plants.

### 3.4 The expression patterns of *LcGRAS* genes during heat, drought, and cold stress

**Figure 5:**
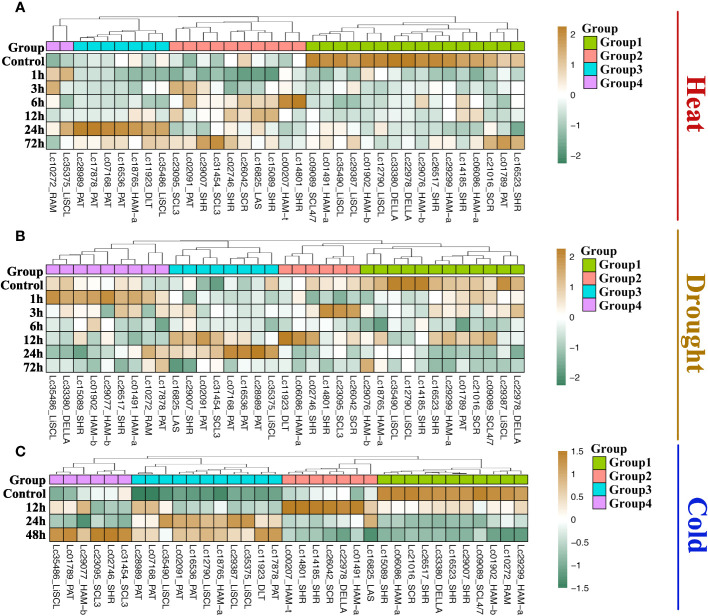
Expression of LCGRAS genes in the treatment with heat **(A)**, drought **(B)** and cold **(C)**. The heatmap shows the mean transcripts per million (TPM) level of 3 biological replicates. Genes whose expression levels were too low were not shown on the figure.

**Figure 6:**
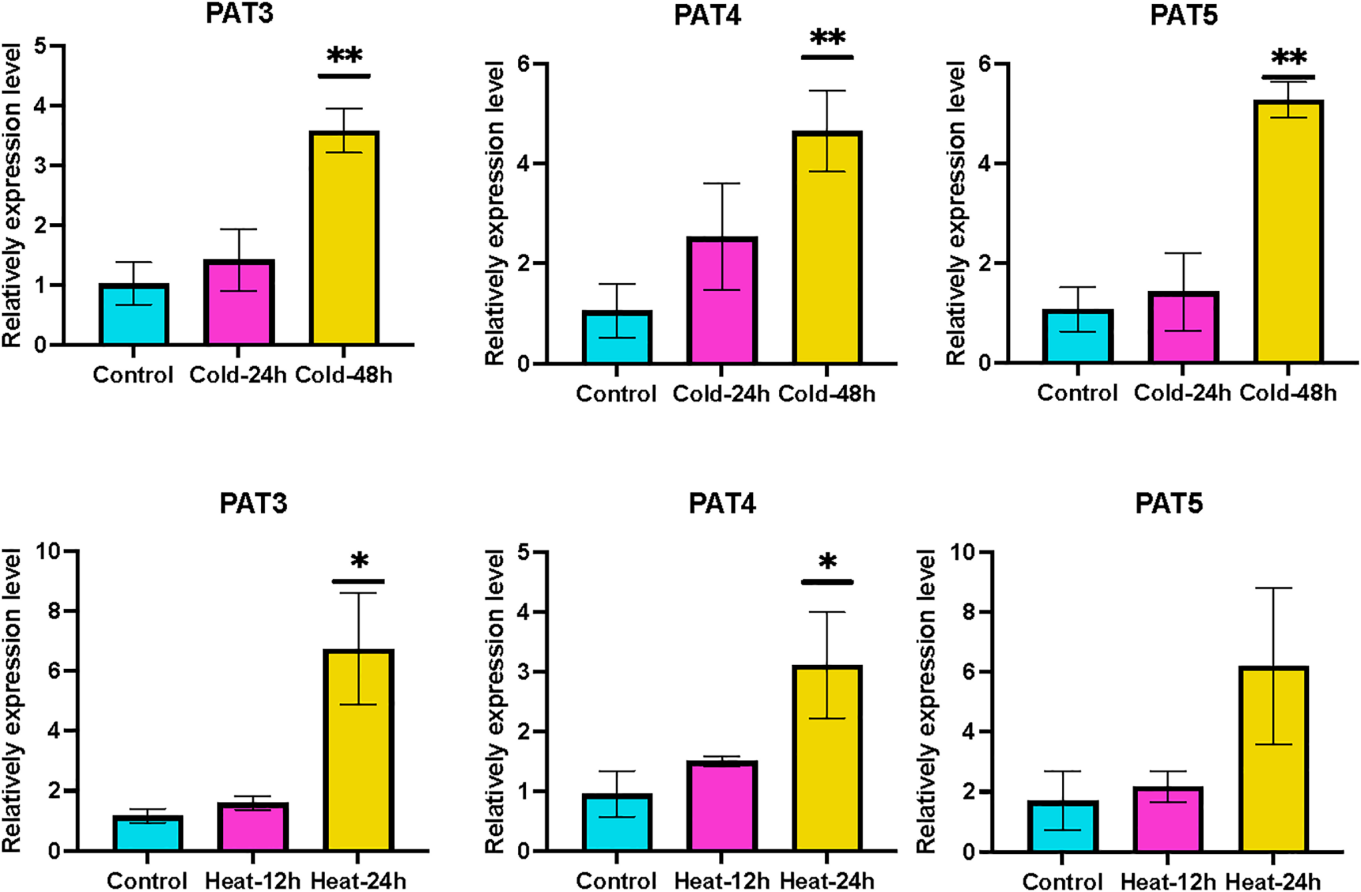
The qRT-PCR results of PATS after cold and hot treatment. Mean value ± SE are shown for the 3 replicates, and the levels of significance relative to the control (without treatment) are analysis by t-test (* represents, p < 0.05; ** represents, p <0.01).

The *GRAS* gene family not only has diverse functions in plant growth but also is responsive to most biotic or abiotic stresses. Heat, drought, and cold are the common stresses for plants in nature. We analyzed the transcriptome data of *Liriodendron* under heat, drought, and cold stress treatments to study the expression pattern of *LcGRAS*. We found that the expression pattern of *LcGRAS* is variable (**Figure 5**). Most genes of *LcGRAS* have changed expression levels within 24 h and 48 h of these three treatments. Some genes, such as *Lc02461* and *Lc34435*, were barely expressed (**Supplementary Table S3**). In heat stress, the genes in Group 1 and Group 4 are sensitive to heat stress, for which their expression levels could change within 1 h (**Figure 5A**). Under drought treatment, *LcGRAS* genes in Group 1, Group 2, and Group 3 were transiently upregulated, and the genes in Group 4 were gradually downregulated (**Figure 5B**). In the cold treatment, most *LcGRAS* genes respond after 12 h, and the expression patterns could be divided into four groups (**Figure 5C**). In Group 1, genes were downregulated and showed persistent low expression over 48 h. The genes in the second group were upregulated at 12 h and were immediately downregulated at subsequent time points. The two other groups were upregulated at 24 h or 48 h. With longer stress treatments (72 h), the expression levels show fluctuation. In contrast, all genes in the PAT subfamily were all upregulated after cold stress, suggesting that PAT genes may not only have a function in phytochrome A signal transduction but can also help plants resist stressful environments. In addition, we selected a subset of genes to verify that the expression level changes were consistent with the transcriptome. The relative expression levels of PAT3, PAT4, and PAT5 by qRT-PCR were similar with transcriptome data (**Supplementary Table S7** and **Figure 6**). With the published papers (Wu et al., 2021; Wu et al., 2022), the studies demonstrate the reliability of transcriptome data.

### 3.5 Overexpression of PAT downregulates *PIF* but upregulates *COR*

**Figure 7:**
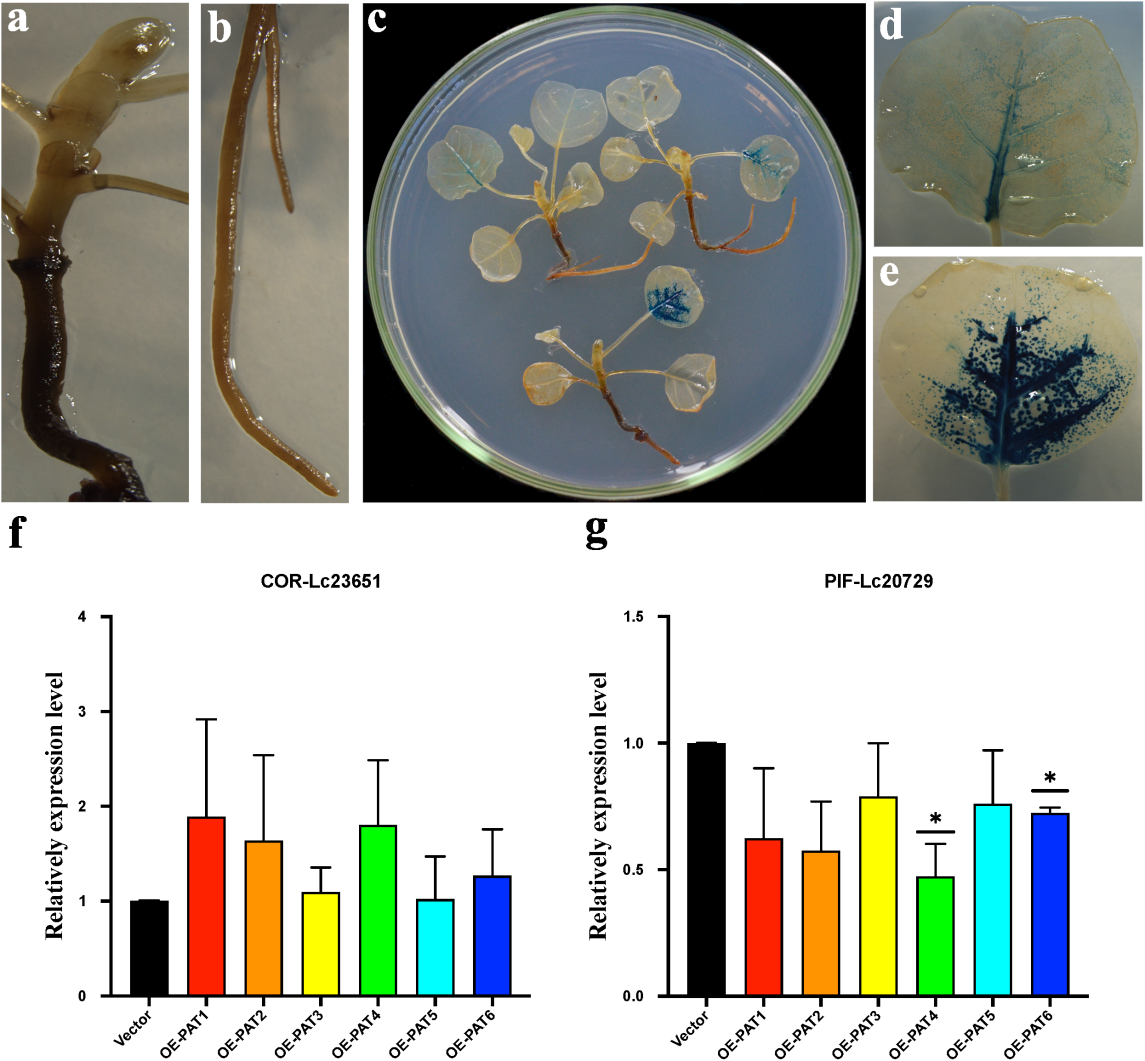
**(A-E)** The GUS stain after transient genetic transformation. **(A)** represents the shoot and steam; **(B)** represents the root; **(C)** represents the whole plants; **(D, E)** represent the leaves. **(F, G)** The relatively expression level of *COR* (*Lc23651*) and *PIF* (*Lc20729*) by qRT-PCR. Mean value ± SE are shown for the 3 replicates, and the levels of significance relative to the control (*35s:GUS*) are analysis by t-test (* represents, p <0.05).

As mentioned above, PAT genes are upregulated after cold treatment. We reasoned that this subfamily may have functions in responding to cold stress. It has been reported that there is complex crosstalk between light and cold signaling pathways (Kidokoro et al., 2022). For example, phytochrome B could enhance cold resistance by promoting the degradation of *PIF* (phytochrome-interacting factor) proteins, which in turn release *COR* (cold-regulated) from the inhibition of PIF (Xu and Deng, 2020). Phytochrome A has also been reported to play a role in acquiring freezing tolerance in *Arabidopsis* (Prerostova et al., 2021). Here, we identified two *PIF* and four *COR* genes in *L. chinense*. Consistently, we noticed that one *PIF* (*Lc20729*) gene and one *COR* (*Lc23651*) gene were strongly regulated under cold stress in our transcriptome data (**Supplementary Table S7**). Additionally, the result of qRT-PCR demonstrated that the expression levels of *PIF* and *COR* changed in accordance with transcriptome data under cold stress (**Supplementary Figure S5**). In order to investigate the function of PAT in cold resistance, we transiently overexpressed six *LcPAT*s (PAT1-PAT6) in *Liriodendron* based on the method developed in *T. hispida* (Zang et al., 2017) and quantified the expression of *COR* and *PIF*. First, we used 35S:GUS as the control to test whether the transient expression system works in *Liriodendron*, and we found that the leaves exhibit strong GUS staining. By contrast, we could not detect clear GUS staining in the root or stem (**Figure 7**). Therefore, we only collected the transformed leaves to analyze the expression levels of *COR* and *PIF* via real-time quantitative PCR (qPCR). *PAT1*–*PAT6* were overexpressed after transformation, although the overexpression level varied among biological replicates (**Supplementary Figure S5**). The sample that was transformed with an empty vector was used as the control. We found that the *COR* was upregulated, and *PIF* was downregulated after overexpressing *PAT*s. *COR* and *PIF* responded to the overexpression of *PAT1* and *PAT4* more actively, but not to *PAT3* and *PAT5*. We could not rule out the possibility that *PAT3* and *PAT5* were lowly expressed after transformations. These results suggest that the cold may stimulate the expression of *PAT* via *PIF* and *COR* to help plants acquire cold tolerance.

### 3.6 The expression pattern of *LcGRAS* genes during somatic embryogenesis and in different tissues in *Liriodendron*

**Figure 8:**
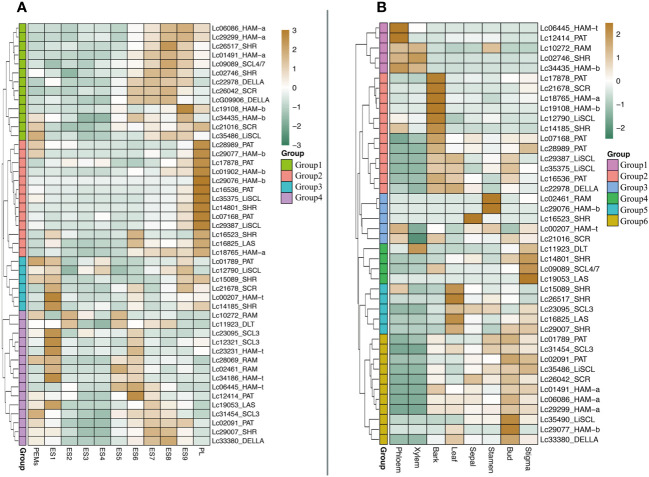
**(A)** The expression of LcGRAS genes during the hybrid Liriodendron somatic embryogenesis. (PEM: embryogenic callus; ES1: after 10 days of liquid culture; ES2: two days after sieving; ES3: one day after ABA treatment; ES4: three days after ABA treatment; ES5: globular embryo; ES6: heart shaped embryo; ES7: torpedo embryo; ES8: immature cotyledon embryo; ES9: mature cotyledon embryo; PL: planta). **(B)** The expression level of LCGRAS in different organs of *L. chinense*.

It is reported that some *GRAS* genes are highly expressed during embryogenesis and in non-differentiated proliferating embryogenic tissue (Abarca et al., 2014). However, whether GRAS genes are expressed during somatic embryogenesis is not known yet. We analyzed the expression level of *LcGRAS* during somatic embryogenesis in *Liriodendron hybrid*. We found that not all *LcGRAS*s are expressed at a high level during somatic embryogenesis. They could be divided into four groups (**Figure 8**; **Supplementary Table S4**). In Group 1 and Group 2, the genes were not expressed until after a heart-shaped embryo developed, which is similar to *GRAS* genes in *Cucumis sativus* (Wiśniewska et al., 2013). The genes in Group 3 and Group 4 were expressed in PEMs or ES1. The majority of *LcGRAS* genes are not expressed in ES3 and ES4. Therefore, LcGRAS may not be involved in the early patterning stages of somatic embryogenesis, but they have a function in late embryo development.

Next, we checked the transcriptome data of different tissues in mature plants (**Figure 8B**; **Supplementary Table S5**). Most *LcGRAS* genes show a strongly specific expression pattern in different tissues. Two of the DELLAs were expressed in all eight tissues, and this indicated that DELLAs are closely related to organ development and produce a long-term effect on plant growth (**Supplementary Table S5**). There are a few genes expressed in the phloem and xylem, but many *LcGRAS*s are expressed in the bark, leaves, bud, and stigma. Interestingly, some *LcGRAS*s have higher expression levels in the stigma than in the stamen, such as *Lc11923*, *Lc01491*, *Lc18765*, *Lc02091*, *Lc09089*, and *Lc29007*. These genes may have key roles in the formation of floral organs.

## 4 Discussion

The GRAS gene family plays a pivotal role in regulating the progression of plant growth and development (Laskar et al., 2021), such as the response to GA signaling and establishing radial root patterning and phytohormone signaling pathways (Guo et al., 2017). The *GRAS* gene family has been identified and analyzed in many model plants, such as *A. thaliana* (Lee et al., 2008), *P. trichocarpa* (Liu and Widmer, 2014), and *Z. mays* (Guo et al., 2017). In addition, their regulation with respect to root and fruit development (Chen et al., 2021) also prompted people to study their function in many economic species, including pepper (*C. annuum* L.) (Liu et al., 2018), apple (*M. domestica*) (Fan et al., 2017), and *Prunus persica* (Jiang et al., 2022). However, the classification of subfamilies is still uncertain, and the number of genes varies greatly among different species. The evolutionary analysis of the *GRAS* gene family and their function requires in-depth exploration. Therefore, we systematically identified and analyzed the *GRAS* gene family in *L. chinense* to provide a basis for further studying the evolution and potential function of the GRAS gene family in tree species.

### 4.1 LcGRAS gene structure and conserved domain analysis

In *L. chinense*, we identified 49 genes that belong to the *GRAS* gene family. The gene number of this gene family is large. There could be more than a hundred members in *G. hirsutum* (Zhang et al., 2018), or there could only be 34 members in *A. thaliana*. The core function of GRAS is closely related to plant development and stress resistance.

Among 49 *LcGRAS*s, we found that *Lc23231* is a short gene with a GRAS domain. The short genes were also identified in *A. thaliana*, *A. trichopoda*, *O. sativa*, and other several species. The alignment result shows that there is no obvious motif that could be found among these short genes (**Supplementary Figure S1**). We found that *Lc23231* has a unique absence of Motif 3 (**Figure 1**), which leads to the sequence terminating prematurely. Similarly, *AT5G67411* (SCL16) is believed to be a putative expressed pseudogene and a unique member of the GRAS family, lacking signature motifs in *A. thaliana* (Lee et al., 2008). However, *Lc23231* still responds to stress treatments and has different expression levels between different tissues in *Liriodendron*. *VviSCL3B* also presents a premature stop codon and lacks motifs PFYRE and SAW, but it was found to be expressed (Grimplet et al., 2016). Therefore, we speculate that *Lc23231* may not be a pseudogene and has functions, which may be due to replication events or structure variation. The delay of *Lc34435* termination was also unexpected. We hypothesized that this was related to retrotransposon insertion. Since *Lc23231* and *Lc34435* are barely expressed in our existing transcripts, determining whether changes in the nucleotide sequence affect their gene function is difficult. This problem requires performing more molecular experiments for proof. In addition, the majority of *LcGRAS* genes (76.3%) were intron-less. This is similar to most results from analyses of other plants, such as *C. annuum* (84%) (Liu et al., 2018), *Hippophae rhamnoides* (58.1%) (Yu et al., 2021), and *Hibiscus hamabo* (62.71%) (Ni et al., 2022). One possible reason is that GRAS genes originate from the bacterial prokaryotic genome (Zhang et al., 2012).

### 4.2 Gene family expansion and evolutionary analysis

Tandem and segmental duplications are the main ways that plants can expand the gene family (Cannon et al., 2004). A large number of duplications was identified in *G. hirsutum* (Li et al., 2018). However, WGD is the main form of duplication in *Liriodendron*, which contributed significantly to gene family expansion for *Liriodendron*.

In general, 49 genes of *LcGRAS* were divided into 13 subgroups, including LiSCL, SHR, PAT, SCL3, RAM, DELLA, DLT, SCR, SCL4/7, LAS, HAM-a, HAM-t, and HAM-b. We distinguished the three subclusters from the cluster of HAM. The members of cluster HAM-t are from woody plants. The following question still requires more data and experiments in order to obtain answers: Is this cluster unique for woody plants or do the genes of HAM-t have a function that is unique for woody plants and does this indicate the evolution traits of woody and herbaceous plants? Collinearity analyses with *L. chinense*, *A. thaliana*, *P. trichocarpa*, and *E. ferox* show that the number and homology of GRAS are species-specific, other than evolutionary status. *L. chinense* has more collinearity blocks with *P. trichocarpa* than *E. ferox*. The evolution of the GRAS gene family was hypothesized to occur after the divergence event (Grosche et al., 2018), and GRAS proteins may play significant roles in complex morphogenetic pattern formation in higher plants such as rootless, leafless thallophytes (Laskar et al., 2021).

### 4.3 The roles of LcGRAS in plant growth, signal transduction, and abiotic stress

We detected the cis-acting regulatory elements in the promotors of *LcGRAS*. Except for gibberellin responsiveness and light responsiveness, we also found cis-acting regulatory elements related to auxin, MeJA, ABA, and stress responsiveness, suggesting that GRAS has complex biological functions. GRAS not only may be involved in signaling pathways for GAs (Peng et al., 1999), phytochrome (Tong and Chu, 2009), and brassinosteroids (BRs) (Silverstone et al., 1998) but also may be involved in other plant hormones such as auxin, MeJA (Hou et al., 2010), and ABA. Their functions of responding to stress and growth may be inextricably linked with the regulator of ABA or auxin. GRAS genes play significant roles in plant growth, production, and defense against abiotic and biotic stresses (Khan et al., 2021). The development stage, tissue, environmental conditions, and genotype affect expression levels (Hirsch and Oldroyd, 2009), and this is consistent with the results of our qRT-PCR and transcriptome data. Moreover, it has been reported that *GRAS* is involved in maintaining the normal development of seeds (Chen et al., 2021). The transcriptome data show that *LcGRAS* is expressed during somatic embryogenesis from the globular embryo stage. This suggests that *LcGRAS* may have similar functions in maintaining embryo development during somatic embryogenesis in *Liriodendron*. Furthermore, nine genes (*Lc01789*, *Lc02091*, *Lc07168*, *Lc16536*, *Lc17878*, *Lc22978*, *Lc31454*, *Lc33380*, and *Lc35375*) were highly expressed and regulated during somatic embryogenesis and under abiotic stress. These genes may respond to stress and participate in development control at the same time. We found that the expression level of *PAT* is upregulated after cold and drought treatment (**Figure 8**). There was a similar discovery with respect to *Vitis vinifera* (Grimplet et al., 2016), which indicated that PAT may have a function during cold and drought stress. We used a quick method to confirm its expression levels in a cold-regulated network. The results show that PAT interacts with the cold regulatory network, but the specific regulatory mode is still unclear. The method of transient genetic transformation is not stable in *Liriodendron*, rendering the results unrepeatable. Stable transformations may be a better method for exploring the gene function of *PAT*s under cold stress in *Liriodendron*.

Overall, differences in the number of *GRAS* gene family members among different species may lead to differences in response to stress or hormones. It is worth further studying the diverse functions of the members of the *GRAS* family.

## 5 Conclusion

In this article, we identified 49 *LcGRAS* genes divided into 13 subgroups and 67 *EfGRAS* divided into 11 subgroups. Here, based on ML phylogenetic analysis with the entire protein sequence of *L. chinense*, *A. thaliana*, *E. ferox*, and four other species, we divided the GRAS gene family into 14 clusters. One of these 14 clusters only contains four genes and cannot be grouped with any other clusters. Thus, we named this cluster as unclassified. In addition, we found a cluster that is only composed of the *GRAS* gene family members of woody species among the species that were used to construct the ML tree. We distinguished this cluster from HAM. As a result, HAM has been divided into three subgroups: HAM-a, HAM-b, and HAM-t. *LcGRAS* responded to the cold, drought, and heat treatments. The expression level of *LcGRAS* was different during the different stages of somatic embryos in *L. chinense*. *LcGRAS*s are conserved and played essential roles in responding to stress treatments and the development of *L. chinense*. In conclusion, these findings provide a basis for further studying the evolution and biological function of the *GRAS* gene family.

## Data availability statement

The original contributions presented in the study are included in the article/**Supplementary Material**. Further inquiries can be directed to the corresponding authors.

## Author contributions

YW, ZH, and JZ provided data collection and analysis methodology; YW, LL, and JC designed the experiments; YW and XC performed the experiments; YW, XW, JW, JC, and JS contributed to the management of this project and manuscript review. All authors contributed to the article and approved the submitted version.
