# Supplementary figures and images for: Genome-wide analysis of the GRAS gene family in Liriodendron chinense reveals the putative function in abiotic stress and plant development
Source: Front Plant Sci. 2023 Sep 21;14:1211853. doi: 10.3389/fpls.2023.1211853 (PMC10551155; doi:10.3389/fpls.2023.1211853)

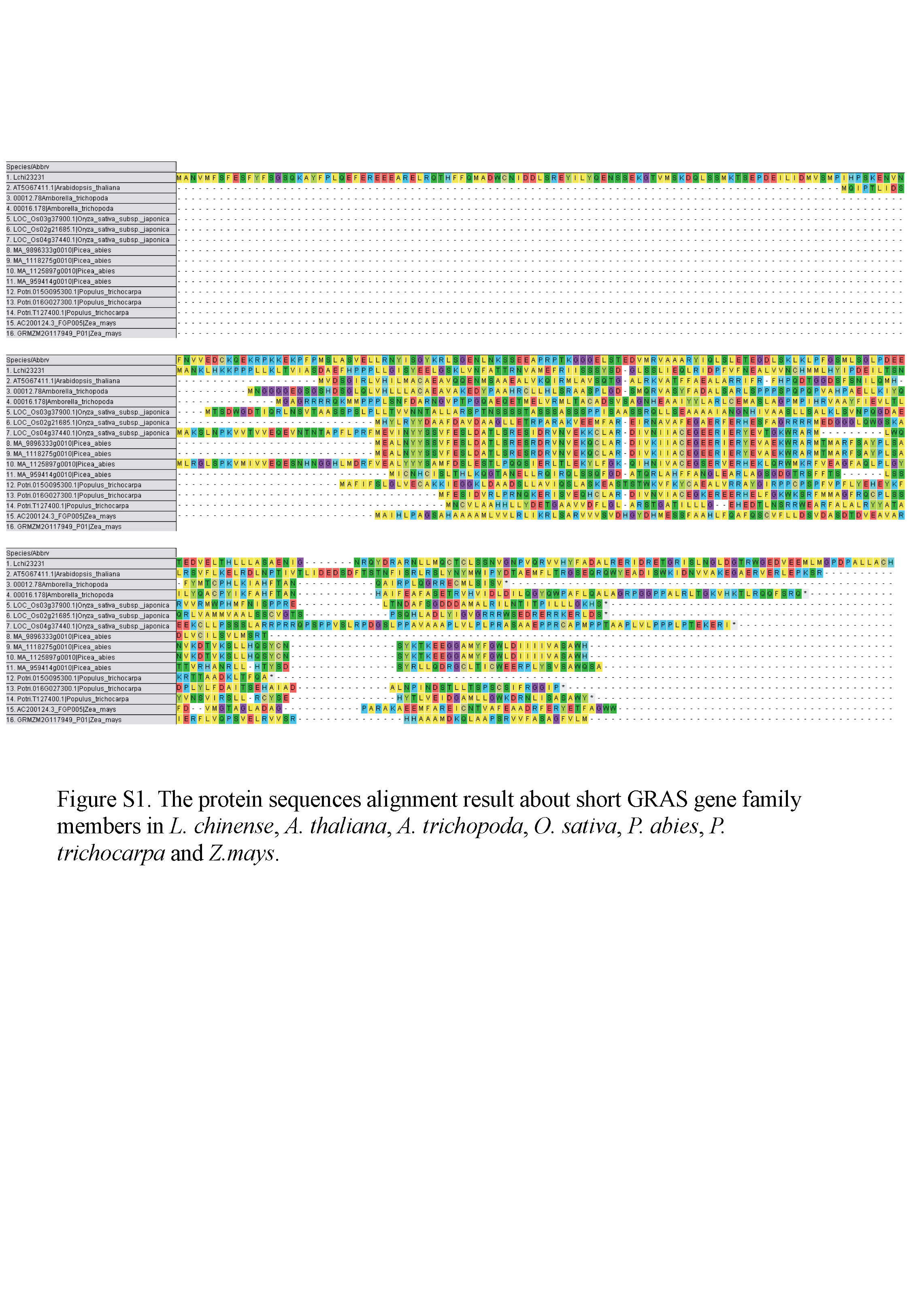

Supplement: Supplementary file 1 [file Image_1.tiff]

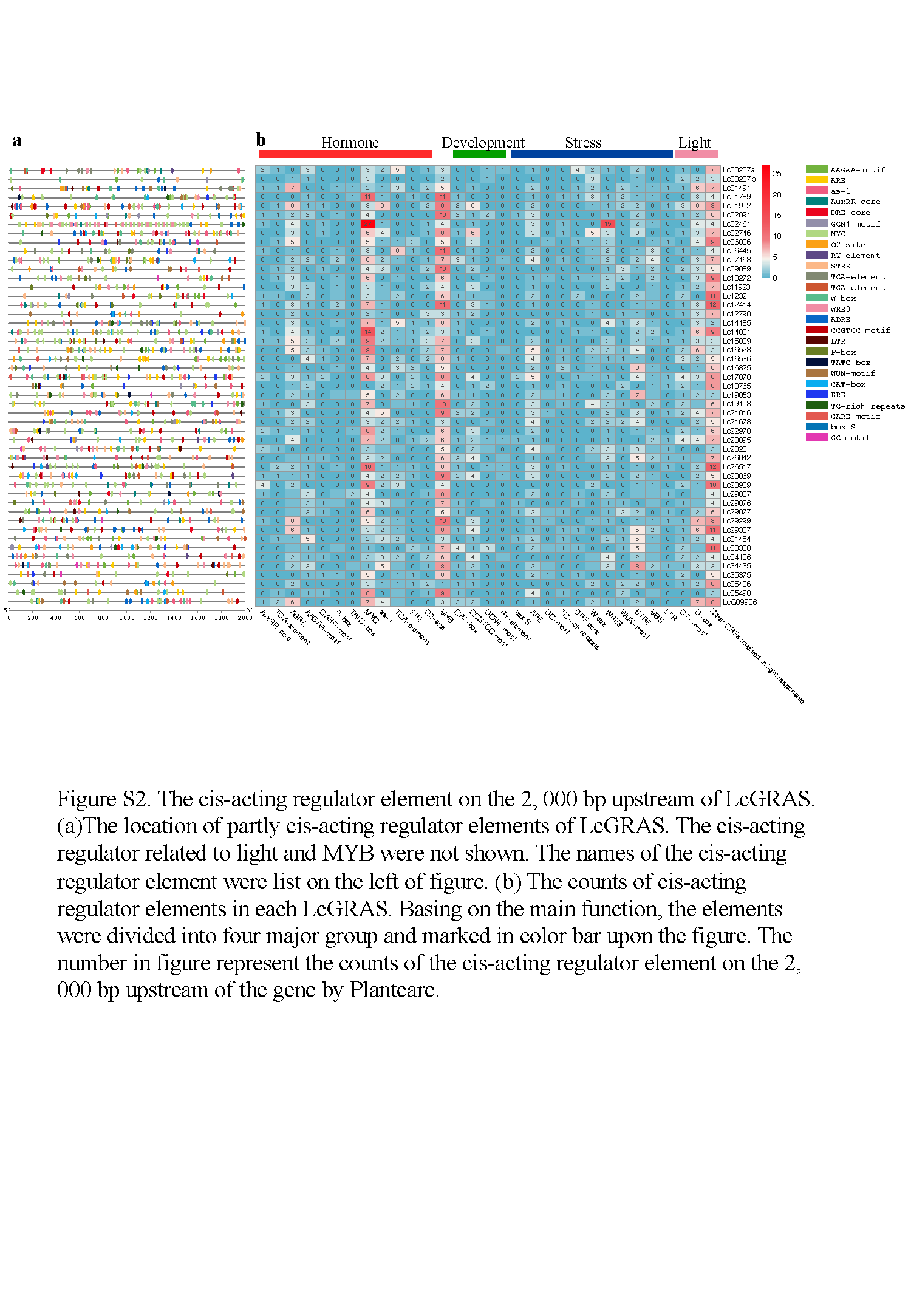

Supplement: Supplementary file 2 [file Image_2.tiff]

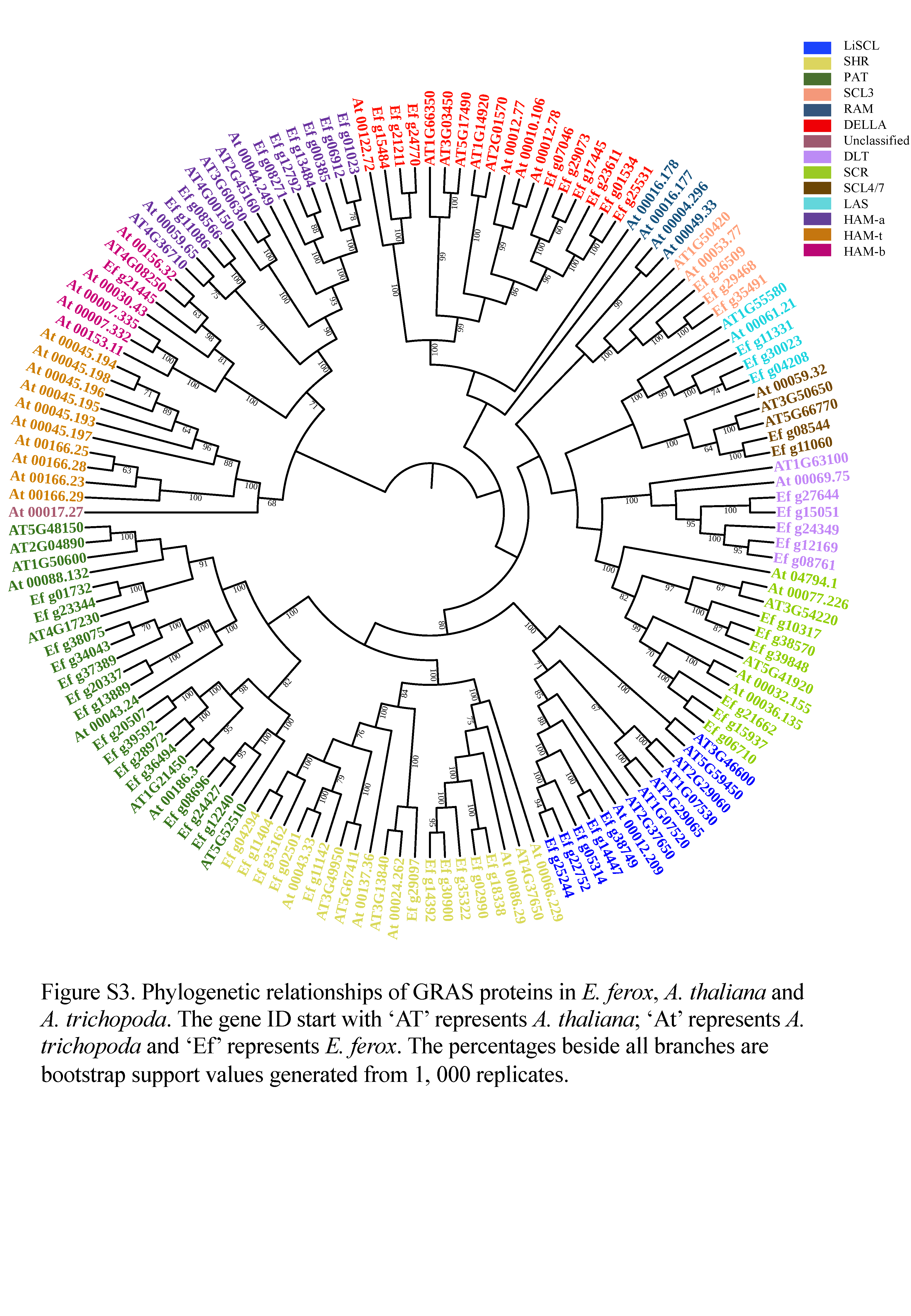

Supplement: Supplementary file 3 [file Image_3.tiff]

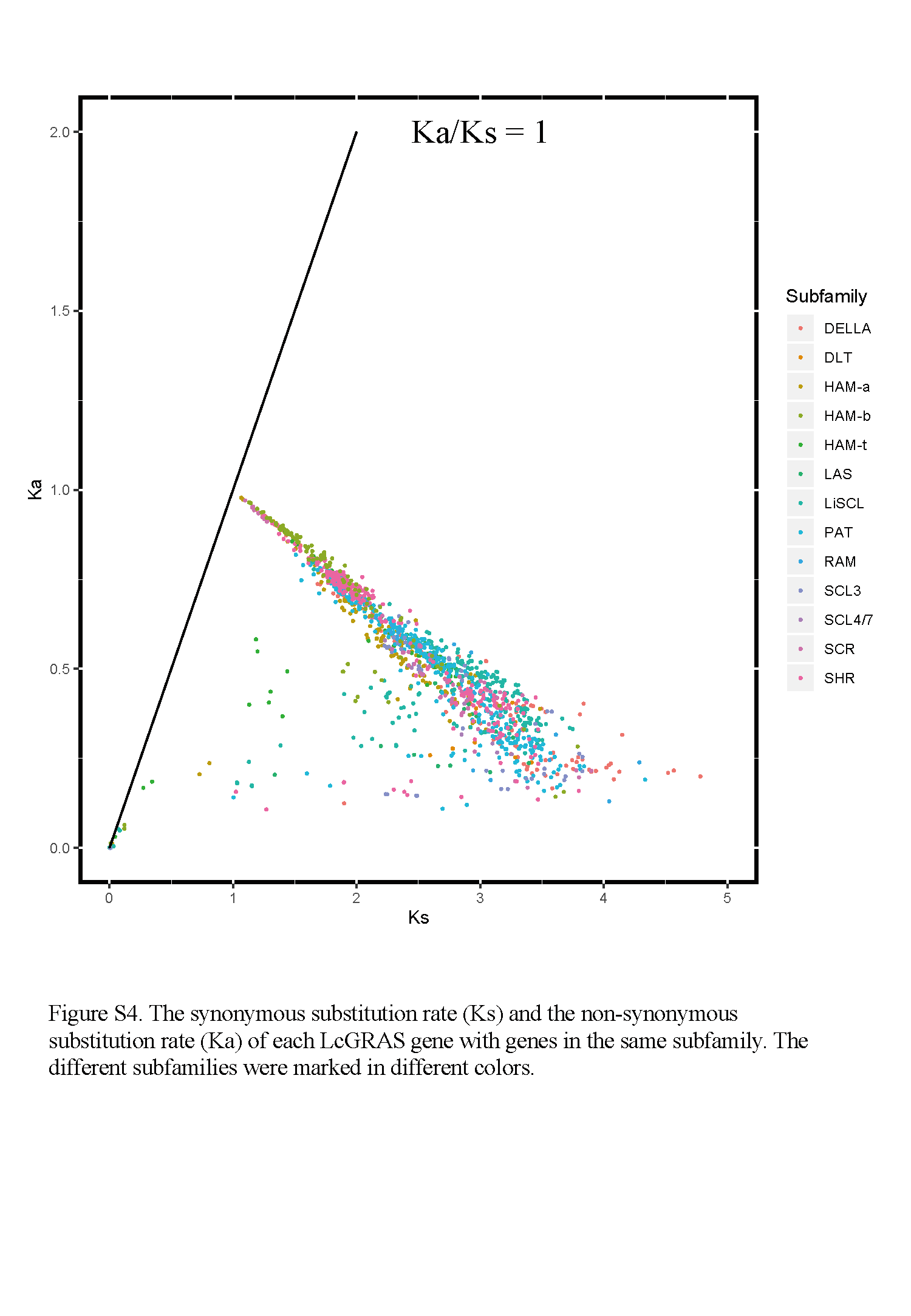

Supplement: Supplementary file 4 [file Image_4.tiff]

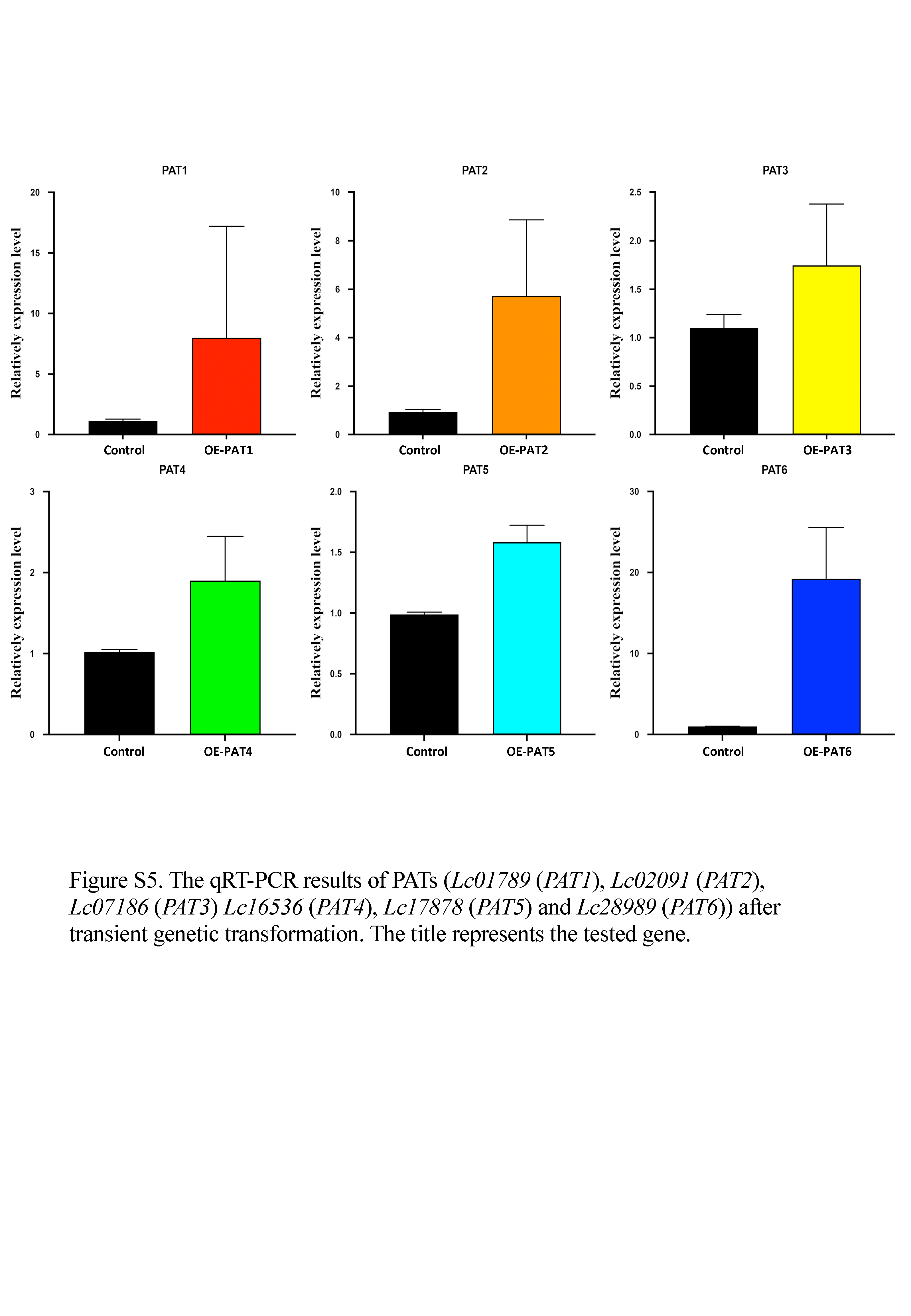

Supplement: Supplementary file 5 [file Image_5.tiff]
